# Historically, did Cemented Thompson perform better than uncemented Austin Moore hemiarthroplasty for femoral neck fractures? A meta-analysis of available evidence

**DOI:** 10.1051/sicotj/2019031

**Published:** 2019-09-06

**Authors:** Mohamed S.A. Shehata, Ahmed Abdelal, Sami Salahia, Hussien Ahmed, Muhammad Shawqi, Ahmed Elsehili, Mahmoud Morsi, Ahmed M. Afifi, Nardeen Kader, Florian Grubhofer, Asser Sallam, Mohamed Imam

**Affiliations:** 1 Faculty of Medicine, Zagazig University 44519 Zagazig Egypt; 2 Medical Research Group of Egypt 44523 Cairo Egypt; 3 Faculty of Medicine, Ain Shams University 11566 Cairo Egypt; 4 Faculty of Medicine, Assuit University 71515 Assuit Egypt; 5 Faculty of Medicine, Menoufia University 32511 Menoufia Egypt; 6 Trauma and Orthopaedics, Ashford and St Peters Hospitals NHS Foundation Trust KT16 0PZ Surrey UK; 7 Department of Orthopaedic surgery, Der Balgrist, University of Zurich 8008 Zurich Switzerland; 8 Department of Orthopaedic Surgery and Trauma, Suez Canal University Hospitals 41522 Ismailia Egypt; 9 Trauma and Orthopaedics, Oxford University Hospitals OX3 9DU Oxford UK

**Keywords:** Femoral neck fractures, Hemiarthroplasty, Thompson, Austin Moore

## Abstract

*Introduction*: Thompson and Austin Moore prostheses have been commonly used in hemiarthroplasties for displaced femoral neck fractures. There has been considerable debate about which of these prostheses is preferred. The purpose of this meta-analysis was to compare historical data for clinical outcomes of cemented Thompson and uncemented Austin Moore hemiarthroplasty in displaced femoral neck fractures.

*Methods*: We searched Medline via PubMed, Cochrane Central, Scopus, Ovid and Web of Science for relevant articles up to February 2019. The included outcomes measured were hip function, hip pain, implant-related complications, surgical complications, reoperation rate and hospital stay. The data were pooled as risk ratio (RR) or mean difference (MD) with 95% confidence interval (CI) between the two compared groups in a meta-analysis model.

*Results*: Ten studies (four RCTs and six observational studies) with a total of 4378 patients were included in the final analysis. The pooled RR showed that the Thompson group was associated with a lower incidence of postoperative hip pain (RR = 0.66, 95% CI [0.54, 0.80]), lesser reoperation rate (RR = 0.46, 95% CI [0.24, 0.88]), lesser intraoperative fractures (RR = 0.15, 95% CI [0.09, 0.25]), but a longer operative time (MD = 12.04 min, 95% CI [2.08, 22.00]) in comparison to the Austin Moore group. The effect estimate did not favour either group in terms of hip function, periprosthetic fractures, prosthetic dislocations, wound infection, mortality and hospital stay.

*Conclusion*: Evidence shows that Thompson hemiarthroplasty is better than Austin Moore hemiarthroplasty in terms of hip pain, reoperation rate and intraoperative fractures. Whereas the postoperative hip function is equivalent, these results could be considered when assessing the outcomes in modern hips.

## Introduction

Femoral neck fractures are among the most serious and frequently occurring injuries in the elderly population, with a high risk of mortality and associated complications [1]. Hemiarthroplasty is considered the treatment of choice for displaced femoral neck fractures in elderly patients; however, the choice of implant used remains controversial. Cemented Thompson and uncemented Austin Moore hemiarthroplasties were the two most common procedures of hemiarthroplasty that were used for displaced femoral neck fractures [2–4].

In 1940, Austin Moore implanted the first vitallium prosthesis to replace the proximal femur, then changed to a straight-stemmed prosthesis in 1950 [5]. Modifications were made to preserve proper neck angle, and the stem was fenestrated in the following years. In the 1950s, Thompson was established for hemiarthroplasty for femoral neck fractures. It was initially operated without cement fixation [6], but over practice has changed to cemented procedure [1]. British orthopaedic surgeons favoured the Thompson prosthesis for treatment of femoral neck fractures [3].

Despite unsatisfactory clinical results, Thompson and Austin Moore undoubtedly have played an important role and remain in regular use within developed countries [7–9]. A controversy exists about the use of cemented Thompson or uncemented Austin Moore prostheses in the femur neck fractures [10]. Previous studies showed good clinical outcomes [11, 12], but others reported significant complications for both prostheses [13, 14]. Many randomized controlled trials (RCTs) have found comparable outcomes between Thompson and Austin Moore implants, including similar levels of post-operative hip pain, reoperation rates, restoration of motion, length of hospital stay and loss of independent predilection sites for fractures [8, 10, 15]. One previous study showed a similarity in survival and failure rates [16].

We designed the current systematic review and meta-analysis to evaluate the clinical outcomes between cemented Thompson and uncemented Austin Moore hemiarthroplasties for displaced femoral neck fractures in the elderly patient population to resolve this controversy.

## Methods

All steps of this systematic review were performed in accordance with the Cochrane handbook of systematic reviews and meta-analysis [17].

### Literature search strategy

We searched Medline via PubMed, Scopus, EBSCO, Cochrane library and Web of Science for relevant articles, using the following keywords**:** “Hemiarthroplasty”, “arthroplasty”, “femoral neck fractures”, “intracapsular hip fractures”, “cemented”, “uncemented” and “cementless”. No restrictions by language, country, or publication date were employed. We also searched the bibliography of eligible studies for relevant articles.

### Eligibility criteria and study selection

We included studies that compared patients with displaced intracapsular femoral neck fractures fixed using cemented Thompson hemiarthroplasty or uncemented Austin Moore hemiarthroplasty. We excluded studies that used prostheses other than Thompson or Austin Moore implants. Studies that involved patients with previous fractures of the same hip or pathological fracture were also excluded. Non-competitive studies, animal studies, duplicate references, case reports, conference abstracts and studies from which data could not be reliably extracted were excluded. We conducted eligibility screening in two steps: step (1) title and abstract screening for matching to the inclusion criteria and step (2) full-text screening for eligibility for meta-analysis. Disagreements were resolved through consensus after discussion.

### Outcomes of interest

We included studies that reported the following outcomes: postoperative hip function, postoperative pain, reoperation and revision rate, implant-related complications (including intraoperative fractures, periprosthetic fractures, dislocations of prosthesis, loosening of prosthesis, wound infection and wound hematoma), surgical complications (including postoperative fractures and postoperative infection), operative details (including operative duration and intraoperative blood loss), hospital stay, medical complications and mortality.

### Data extraction

Three independent reviewers extracted the author name, year of publication, study design, number of participants in each group, age, gender, type of intervention (including the type of prosthesis), study period, follow-up period and relevant outcome data. Another reviewer resolved disagreements.

### Risk of bias assessment

For RCTs, two independent reviewers used the Cochrane risk of bias (ROB) assessment tool of the Cochrane handbook of systematic reviews of interventions 5.1.0 [17]. For observational studies, the Newcastle Ottawa scale (NOS) was used [18] which considers selection of population, comparability of groups on demographic characteristics and potential confounders, and ascertainment of the prespecified outcome (exposure/treatment).

### Data analysis

We calculated the risk ratio (RR) and 95% confidence intervals (CI) for dichotomous data, and mean difference (MD) or standardized mean difference (SMD) and 95% CI for continuous data. A value of *p* < 0.05 was considered statistically significant. Missing standard deviation (SD) data were calculated from the equations provided by Altman [19]. Data analysis was conducted using Comprehensive meta-analysis software.

### Assessment of Heterogeneity

Heterogeneity was evaluated by the forest plot methods and measured by Q statistic and *I* ^2^ statistic. Significant statistical heterogeneity was indicated by Q statistic *p*-value less than 0.1 or by *I* ^2^ more than 50%. In case of significant heterogeneity, a random effects model was employed. Otherwise, the fixed effects model was used. We conducted subgroup and sensitivity analyses.

### Publication bias

According to Egger’s and colleagues [20, 21], publication bias assessment is not reliable for less than ten pooled studies per outcome. Therefore, we could not assess the existence of publication bias using Egger’s funnel plot asymmetry.

## Results

### Demographics and characteristics

**Figure 1:**
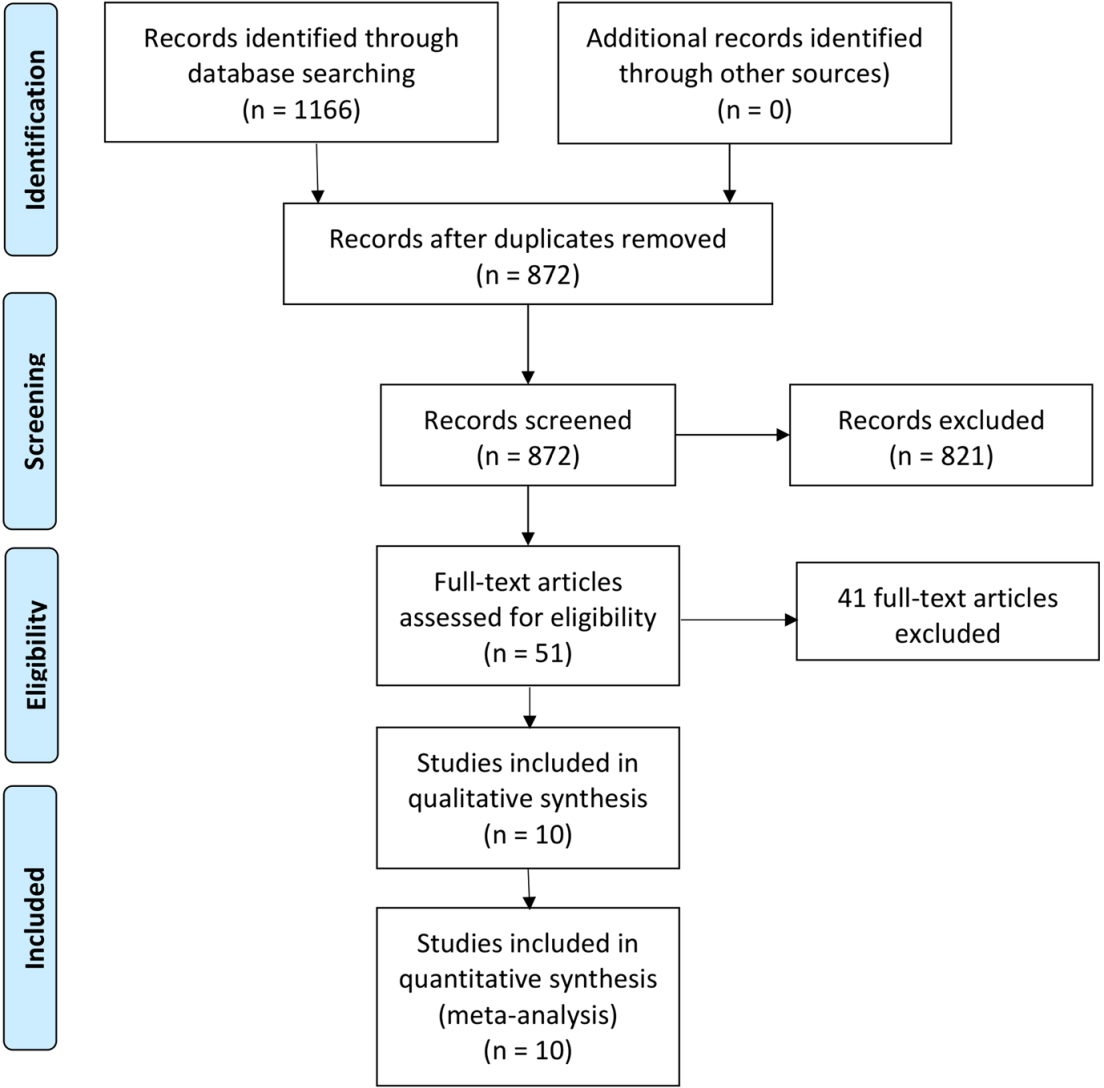
Flow diagram of articles selection process.

Our search retrieved 1166 unique citations. Fifty-one articles were retrieved and screened for eligibility to the meta-analysis. Of them, 41 articles were excluded and 10 articles (four RCTs and six observational studies) were included in the present meta-analysis. Figure 1 shows the study selection process.

### Baseline characteristics and risk of bias assessment

**Figure 2:**
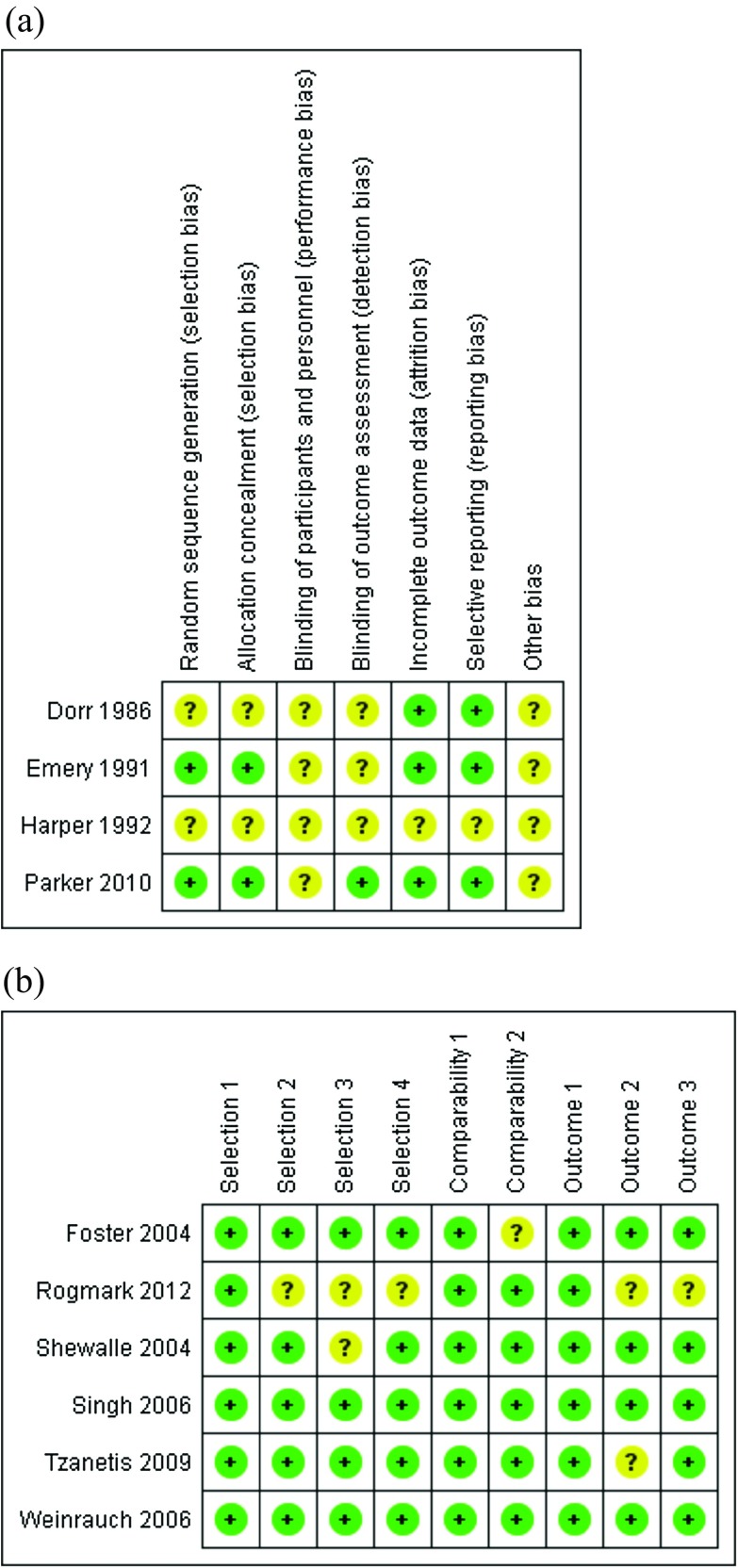
(a) Risk of bias summary of randomized clinical trials; (b) Risk of bias summary of observational studies.

Ten studies [1, 8, 10, 15, 22–27] (four RCTs and six observational studies) were included in the final analysis investigating a total of 4378 participants: of them, 2632 were treated by the Thompson prosthesis and 1746 were treated by the Austin Moore prosthesis. The follow-up ranged from 1 year to 8 years. All studies were published in English from 1986 to 2012. The summary of the included studies and baseline characteristics of their population are shown in Table 1. The quality of the included studies ranged from moderate to high, Figure 2.

**Table 1:** Baseline characteristic of included studies.

Study ID	Study design	Number of patients		Age		Gender, female		Intervention	Study period	Follow-up in months
		TP	AMP	TP	AMP	TP	AMP			
Rogmark et al. [15]	Observational study	1116 (64%)	616 (36%)	80		826 (74%)	462 (75%)	TP/AMP HA	2005–2009	12
Tzanetis et al. [26]	Observational study	137 (36%)	245 (64%)	79	77.5	103 (75%)	196 (80%)	TP/AMP HA	1986–1977	36/96
Parker et al. [23]	RCT	200 (50%)	200 (50%)	83	83	161 (80%)	147 (73%)	TP/AMP HA	2001–2006	6
Weinrauch et al. [27]	Observational study	738 (66%)	380 (34%)	NA		NA		TP/AMP HA	1998–2003	32/36
Singh and Deshmukh [1]	Observational study	25 (64%)	29 (54%)	84	83	21 (84%)	25 (86.2%)	TP/AMP HA	1999–2000	12
Foster et al. [24]	Observational study	174 (71%)	70 (29%)	80	83	138 (79.3%)	52 (74.28%)	TP/AMP HA	2001–2002	NA
Shewale et al. [25]	Observational study	100 (50%)	100 (50%)	84.3	85.4	87 (87%)	87 (87%)	TP/AMP HA	Over two years	24
Harper.and Gregg [2]	RCT	77 (54%)	66 (46%)	83		143 (100%)		TP/AMP HA	NA	12
Emery et al. [10]	RCT	27 (51%)	26 (49%)	78	79.6	24 (89%)	22 (85%)	TP/AMP HA	NA	17
Dorr et al. [8]	RCT	37 (74%)	13 (26%)	72	66	26 (70%)	9 (69%)	TP/AMP HA	1980–1982	24/48

AMP, Austin Moore prosthesis; HA, hemiarthroplasty; NA, not available; RCT, randomized controlled trials; TP, Thompson prosthesis.

### Outcomes

#### Postoperative hip function

**Figure 3:**
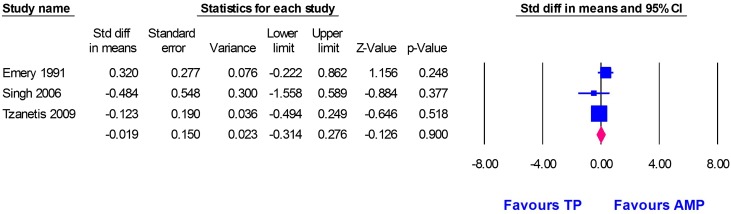
Forest plot of standardized mean difference (SMD) in functional scores with 95% confidence interval, comparing Thompson and Austin Moore groups.

Three studies [1, 10, 26] reported data on HHS, with 189 patients in the Thompson group and 300 patients in the Austin Moore group. The pooled SMD did not favour either group in hip function (SMD = −0.02, 95% CI [−0.31, 0.28], *p* = 0.9). No substantial evidence of heterogeneity was noted (*I* ^2^ = 20.61%, *p* = 0.28), Figure 3.

### Postoperative pain

**Figure 4:**
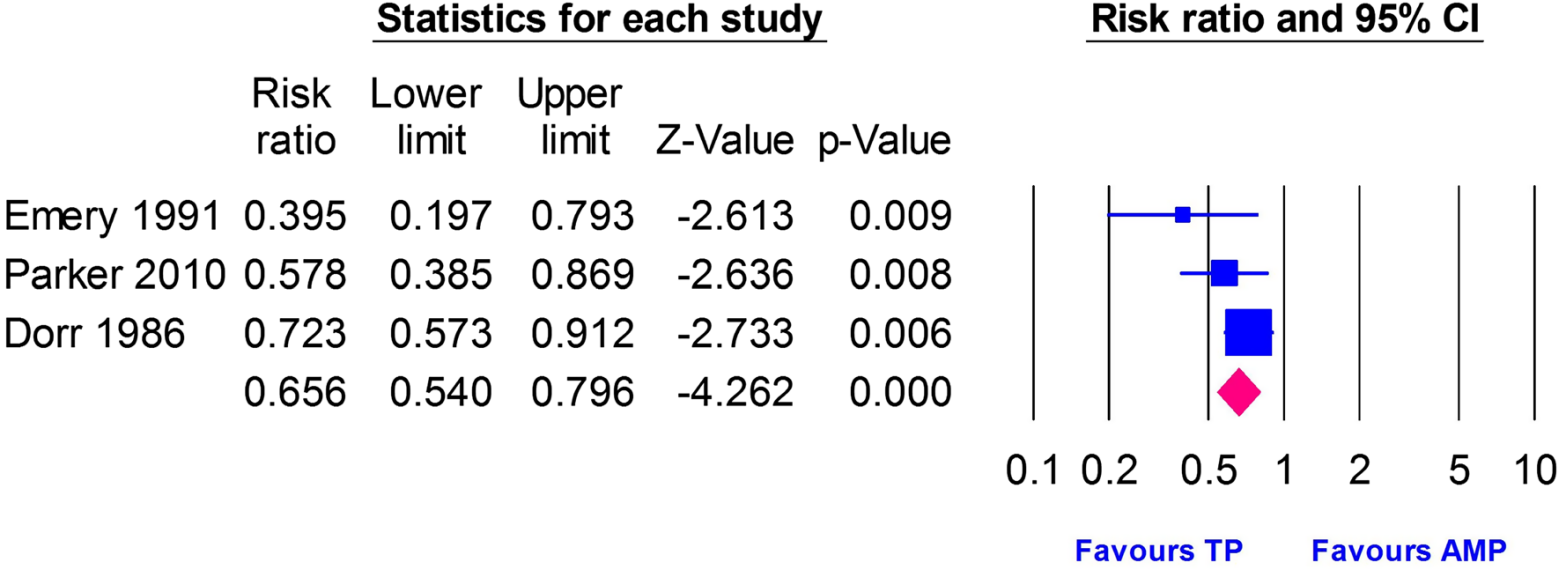
Forest plot of risk ratio (RR) of postoperative pain with 95% confidence interval, comparing Thompson and Austin Moore groups.

Three studies [8, 10, 23] reported data on postoperative pain, with 264 patients in the Thompson group and 239 patients in the Austin Moore group. The pooled RR showed that the Thompson group was associated with a lower incidence of postoperative pain than the Austin Moore group (RR = 0.66, 95% CI [0.54, 0.80], *p* < 0.0001). There was no significant heterogeneity (*I* ^2^ = 35.17%, *p* = 0.21), Figure 4.

### Reoperation and revision rate

**Figure 5:**
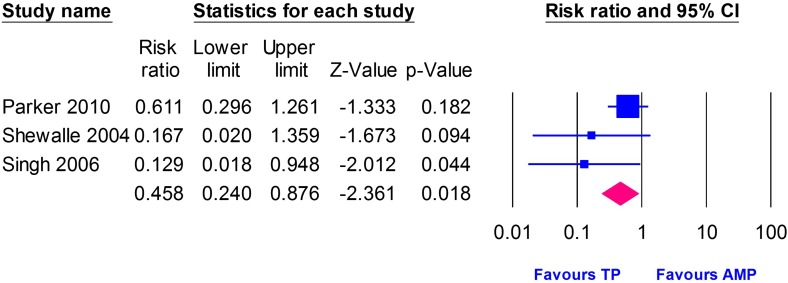
Forest plot of risk ratio (RR) of reoperation and revision rate with 95% confidence interval, comparing Thompson and Austin Moore groups.

Three studies [1, 23, 25] provided data on reoperation and revision rate, including 325 patients in the Thompson group and 329 patients in the Austin Moore group. The pooled estimate showed significantly lower reoperation and revision rates in the Thompson group compared to the Austin Moore group (RR = 0.46, 95% CI [0.24, 0.88], *p* = 0.02). Pooled studies were homogenous (*I* ^2^ = 34.46%, *p* = 0.22), Figure 5.

### Implant-related complications

The pooled RR showed that the Thompson group (1323 patients) was related to a lower incidence of intraoperative fractures than the Austin Moore group (1366 patients) (RR = 0.15, 95% CI [0.09, 0.25], *p* < 0.0001, *I* ^2^ = 35,60%; *p* = 0.18), while the two groups were comparable in terms of periprosthetic fractures (RR = 0.22, 95% CI [0.006, 8.67], *p* = 0.42, *I* ^2^ = 65.4%; *p* = 0.09), dislocations of prosthesis (RR = 0.65, 95% CI [0.39, 1.08], *p* = 0.096, *I* ^2^ = 0%; *p* = 0.85) and wound infection (RR = 1.04, 95% CI [0.56, 1.92], *p* = 0.91, *I* ^2^ = 0%; *p* = 0.82), Supplementary material.

### Surgical complications

Two studies [15, 24] provided the results of surgical complications, enrolling 912 patients in the Thompson group and 450 patients in the Austin Moore group. The effect estimate showed no statistically significant difference between the compared groups (RR = 0.22, 95% CI [0.05, 1.03], *p* = 0.06). Pooled studies were homogenous (*I* ^2^ = 9.35%, *p* = 0.29), Supplementary material.

### Operative details

The MD showed that the Thompson group (327 patients) had a longer operative time than the Austin Moore group (326 patients) (MD = 12.04 min, 95% CI [2.08, 22.00], *p* = 0.02, *I* ^2^ = 90.6%; *p* = 0). The MD showed no statistically significant difference between the Thompson and Austin Moore prosthesis groups in terms of hospital stay (MD = −1.42 days, 95% CI [−4.95, 2.10], *p* = 0.43), Supplementary material.

### Medical complications

Two studies reported on medical complications (including pulmonary embolism, cardiac complications and deep venous thrombosis), enrolling 337 patients in the Thompson group and 445 patients in the Austin Moore group. The pooled RR did not favour either group (RR = 0.72, 95% CI [0.42, 1.22], *p* = 0.22). Combined studies were homogenous (*I* ^2^ = 0%, *p* = 0.63), Supplementary material.

### Mortality

Four studies [2, 10, 23, 25] reported on mortality at perioperative, postoperative three months and one year, enrolling 404 patients in the Thompson group and 392 patients in the Austin Moore group. The pooled RR did not favour either group (RR = 0.89, 95% CI [0.72, 1.11], *p* = 0.29). Combined studies were homogenous (*I* ^2^ = 0%, *p* = 0.98), Supplementary material.

## Discussion

Femoral neck fracture is one of the leading causes of mortality in the elderly [28]. While hemiarthroplasty is considered the mainstay treatment for displaced femoral neck fracture [29], there is still ongoing debate regarding the type of prosthesis used. Two types of prosthesis have been predominantly used: the cemented Thompson prosthesis and the uncemented Austin Moore prosthesis. This study aimed to compare the clinical outcomes of both prostheses using a meta-analytic approach of 10 studies; four randomized controlled trials and six observational studies with a total of 4378 patients.

Our study showed that no significant difference existed in hip function between the two groups. Besides, our analysis did not favour Thompson or Austin Moore hemiarthroplasty with regard to mortality figures or medical complications. Our results came in line with another systematic review that compared cemented versus uncemented prosthesis [30, 31]. These results were supported by single studies as well [9, 23, 32, 33], while Parvizi et al. reported an association between the cemented Thompson hemiarthroplasty and cardiopulmonary complications that may lead to sudden death [34].

With regard to operative outcomes, our results indicated that the Thompson group had a lower incidence of surgical complications; however, this was not statistically significant. These findings are in agreement with two previous systematic reviews that compared cemented versus uncemented prosthesis [30, 35]. One operative outcome that yielded a significant difference was intraoperative fractures, with a lower incidence found in the Thompson group; this could be attributed to the wide stem of Austin Moore [25]. An important factor in deciding prosthesis choice is operative time and postoperative hospital stay, especially as the prevalent demographic for this intervention is the elderly population. Our results showed a longer operative time in the Thompson group. The two groups were similar regarding the length of hospital stay. Only one study reported a higher incidence of postoperative blood loss with the Thompson group over the Austin Moore group [10].

Concerning postoperative outcomes, our results showed that the Austin Moore had a higher reoperation rate than the Thompson technique. This was affected by several factors including but not limited to: postoperative pain (which we found to be higher in the Austin Moore group) and prosthetic loosening (Rogmark et al. reported one case of prosthetic loosening related to the Thompson group that required reoperation) [15]. There was no significant difference between the two groups in wound infection; however, one study reported that the Thompson group had a higher incidence of wound hematoma over the Austin Moore group [23]. The two groups were similar in terms of periprosthetic fractures and prosthetic dislocations.

Accordingly, we conclude that the cemented Thompson group was associated with a lower incidence of hip pain and reoperation rate. However, no evidence for a decisive detrimental effect exists. This was in agreement with eight of our studies [1, 10, 15, 23–25, 27], while only Tzanetis et al. and Door et al. gave no preferred choice.

We conducted a comprehensive database search that yielded a great number of high-quality RCTs and observational studies, used a rigorous screening process that allowed us to focus only on the studies that met our selection criteria and were appropriate to our research question. The large sample size (4378 patients) may allow for data generalization application. This is due to our inclusion of observational studies as well as RCTs. Some of our results showed significant heterogeneity, which was best resolved using subgroup and sensitivity analyses. We used the Cochrane Collaboration tool to assess the risk of bias of the included RCTs. For observational studies, we used the Newcastle Ottawa scale. The results of this study are subject to limitations inherent to any meta-analysis based on pooling of data from different trials with various study protocols, different baseline patient characteristics and definitions for efficacy/safety outcomes. The number of studies in each outcome was low, and this could have an impact on the interpretation of the results. Further, only published data were used.

## Conclusion

Available evidence demonstrates that Thompson hemiarthroplasty is better than Austin Moore hemiarthroplasty in terms of hip pain, reoperation rate and intraoperative fractures. In institutions where these prostheses are still used, our results recommend the utilization of Thompson hemiarthroplasty.

## Supplementary Material

**Figure A.1**. Forest Plot of risk ratio (RR) of intraoperative fractures with 95% confidence interval, comparing between Thompson and Austin Moore groups.
**Figure A.2**. Forest Plot of risk ratio (RR) of periprosthetic fractures with 95% confidence interval, comparing between Thompson and Austin Moore groups.
**Figure A.3**. Forest Plot of risk ratio (RR) of prosthetic dislocations with 95% confidence interval, comparing between Thompson and Austin Moore groups.
**Figure A.4**. Forest Plot of risk ratio (RR) of wound infection with 95% confidence interval, comparing between Thompson and Austin Moore groups.
**Figure A.5**. Forest Plot of risk ratio (RR) of surgical complications with 95% confidence interval, comparing between Thompson and Austin Moore groups.
**Figure A.6**. Forest Plot of mean difference (MD) of operative time with 95% confidence interval, comparing between Thompson and Austin Moore groups.
**Figure A.7**. Forest Plot of mean difference (MD) of hospital stay with 95% confidence interval, comparing between Thompson and Austin Moore groups.
**Figure A.8**. Forest Plot of risk ratio (RR) of medical complications with 95% confidence interval, comparing between Thompson and Austin Moore groups.
**Figure A.9**. Forest Plot of risk ratio (RR) of mortality with 95% confidence interval, comparing between Thompson and Austin Moore groups. Supplementary material is available at https://www.sicot-j.org/10.1051/sicotj/2019031/olm

## Conflicts of interest

The authors have no conflicts of interest to declare.
