# Corrigendum to: Historically, did Cemented Thompson perform better than uncemented Austin Moore hemiarthroplasty for femoral neck fractures? A meta-analysis of available evidence

**DOI:** 10.1051/sicotj/2026013

**Published:** 2026-04-16

**Authors:** Mohamed S.A. Shehata, Ahmed Abdelal, Sami Salahia, Hussien Ahmed, Muhammad Shawqi, Ahmed Elsehili, Mahmoud Morsi, Ahmed M. Afifi, Nardeen Kader, Florian Grubhofer, Asser Sallam, Mohamed Imam

**Affiliations:** 1 Faculty of Medicine, Zagazig University 44519 Zagazig Egypt; 2 Medical Research Group of Egypt 44523 Cairo Egypt; 3 Faculty of Medicine, Ain Shams University 11566 Cairo Egypt; 4 Faculty of Medicine, Assuit University 71515 Assuit Egypt; 5 Faculty of Medicine, Menoufia University 32511 Menoufia Egypt; 6 Trauma and Orthopaedics, Ashford and St Peters Hospitals NHS Foundation Trust KT16 0PZ Surrey UK; 7 Department of Orthopaedic surgery, Der Balgrist, University of Zurich 8008 Zurich Switzerland; 8 Department of Orthopaedic Surgery and Trauma, Suez Canal University Hospitals 41522 Ismailia Egypt; 9 Trauma and Orthopaedics, Oxford University Hospitals OX3 9DU Oxford UK

In the published article, errors were identified in the author affiliations. The correct affiliations for Muhammad Shawqi are 2 (Medical Research Group of Egypt, 44523 Cairo, Egypt) and 4 (Faculty of Medicine, Assiut University, 71515 Assiut, Egypt), instead of 2 and 5. The correct affiliation for Sami Salahia is 3 (Faculty of Medicine, Ain Shams University, 11566 Cairo, Egypt), instead of 4.

