# Supplementary material for: Historically, did Cemented Thompson perform better than uncemented Austin Moore hemiarthroplasty for femoral neck fractures? A meta-analysis of available evidence
Source: SICOT J. 2019 Sep 6;5:33. doi: 10.1051/sicotj/2019031 (PMC6753858; doi:10.1051/sicotj/2019031)
Supplement: Supplementary file 1 — Figure A.1. Forest Plot of risk ratio (RR) of intraoperative fractures with 95% confidence interval, comparing between Thompson and Austin Moore groups. Figure A.2. Forest Plot of risk ratio (RR) of periprosthetic fractures with 95% confidence interval, comparing between Thompson and Austin Moore groups. Figure A.3. Forest Plot of risk ratio (RR) of prosthetic dislocations with 95% confidence interval, comparing between Thompson and Austin Moore groups. Figure A.4. Forest Plot of risk ratio (RR) of wound infection with 95% confidence interval, comparing between Thompson and Austin Moore groups. Figure A.5. Forest Plot of risk ratio (RR) of surgical complications with 95% confidence interval, comparing between Thompson and Austin Moore groups. Figure A.6. Forest Plot of mean difference (MD) of operative time with 95% confidence interval, comparing between Thompson and Austin Moore groups. Figure A.7. Forest Plot of mean difference (MD) of hospital stay with 95% confidence interval, comparing between Thompson and Austin Moore groups. Figure A.8. Forest Plot of risk ratio (RR) of medical complications with 95% confidence interval, comparing between Thompson and Austin Moore groups. Figure A.9. Forest Plot of risk ratio (RR) of mortality with 95% confidence interval, comparing between Thompson and Austin Moore groups. [file sicotj-5-33-s1.pdf]

Supplementary material

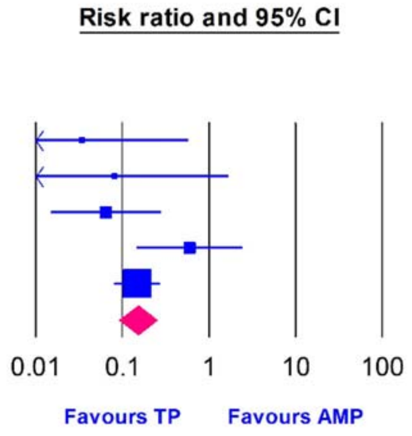

Study name	Risk ratio	Lower limit	Upper limit	Z-Value	p-Value
Parker 2010	0.034	0.002	0.574	-2.347	0.019
Foster 2004	0.081	0.004	1.669	-1.628	0.104
Rogmark 2012	0.065	0.015	0.280	-3.666	0.000
Shewalle 2004	0.600	0.147	2.444	-0.713	0.476
Weinrauch 2006	0.149	0.081	0.272	-6.177	0.000
	0.151	0.091	0.250	-7.352	0.000

Figure A.1. Forest Plot of risk ratio (RR) of intraoperative fractures with 95% confidence interval, comparing between Thompson and Austin Moore groups.

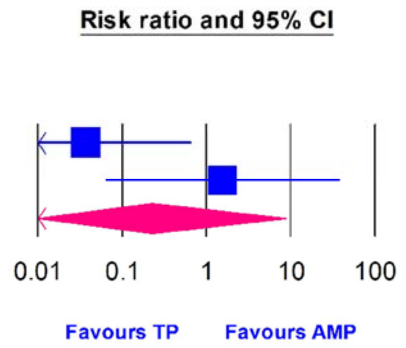

Study name	Risk ratio	Lower limit	Upper limit	Z-Value	p-Value
Foster 2004	0.037	0.002	0.658	-2.244	0.025
Weinrauch 2006	1.547	0.063	37.877	0.267	0.789
	0.223	0.006	8.671	-0.803	0.422

Figure A.2. Forest Plot of risk ratio (RR) of periprosthetic fractures with 95% confidence interval, comparing between Thompson and Austin Moore groups.

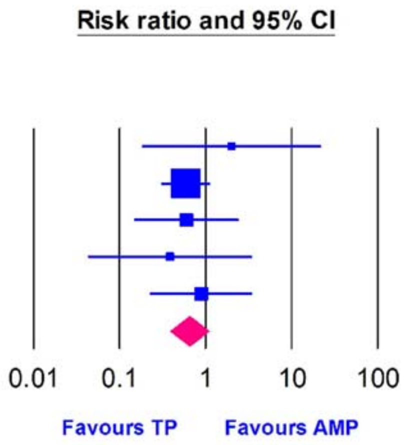

Study name	Risk ratio	Lower limit	Upper limit	Z-Value	p-Value
Parker 2010	2.000	0.183	21.880	0.568	0.570
Rogmark 2012	0.584	0.303	1.126	-1.606	0.108
Shewalle 2004	0.600	0.147	2.444	-0.713	0.476
Singh 2006	0.387	0.043	3.486	-0.847	0.397
Tzanetis 2009	0.891	0.226	3.508	-0.165	0.869
	0.645	0.385	1.081	-1.666	0.096

Figure A.3. Forest Plot of risk ratio (RR) of prosthetic dislocations with 95% confidence interval, comparing between Thompson and Austin Moore groups.

Study name	Risk ratio	Lower limit	Upper limit	Z-Value	p-Value
Emery 1991	2.893	0.123	67.958	0.660	0.510
Parker 2010	1.250	0.504	3.102	0.481	0.630
Shewalle 2004	0.833	0.263	2.642	-0.310	0.757
Tzanetis 2009	0.764	0.201	2.907	-0.395	0.693
	1.035	0.558	1.919	0.108	0.914

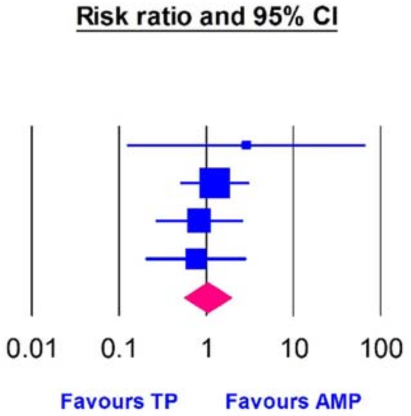

Figure A.4. Forest Plot of risk ratio (RR) of wound infection with 95% confidence interval, comparing between Thompson and Austin Moore groups.

Study name	Risk ratio	Lower limit	Upper limit	Z-Value	p-Value
Foster 2004	0.058	0.003	1.108	-1.892	0.058
Rogmark 2012	0.368	0.062	2.196	-1.097	0.273
	0.224	0.049	1.033	-1.918	0.055

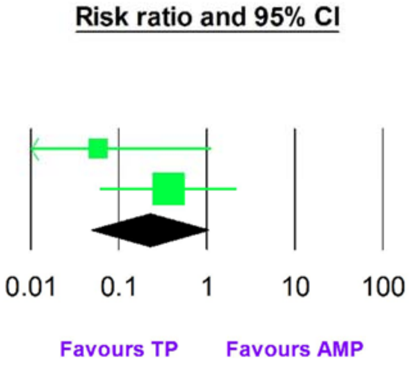

Figure A.5. Forest Plot of risk ratio (RR) of surgical complications with 95% confidence interval, comparing between Thompson and Austin Moore groups.

Study name	Difference in means	Standard error	Variance	Lower limit	Upper limit	Z-Value	p-Value
Emery 1991	8.000	4.732	22.393	-1.275	17.275	1.691	0.091
Parker 2010	6.900	1.330	1.769	4.293	9.507	5.188	0.000
Shewalle 2004	21.000	2.765	7.645	15.581	26.419	7.595	0.000
	12.042	5.081	25.821	2.083	22.002	2.370	0.018

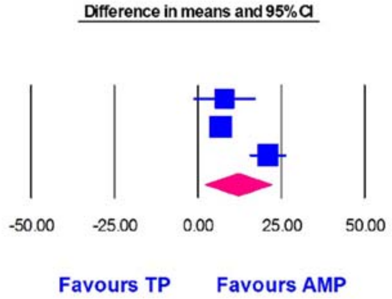

Figure A.6. Forest Plot of mean difference (MD) of operative time with 95% confidence interval, comparing between Thompson and Austin Moore groups.

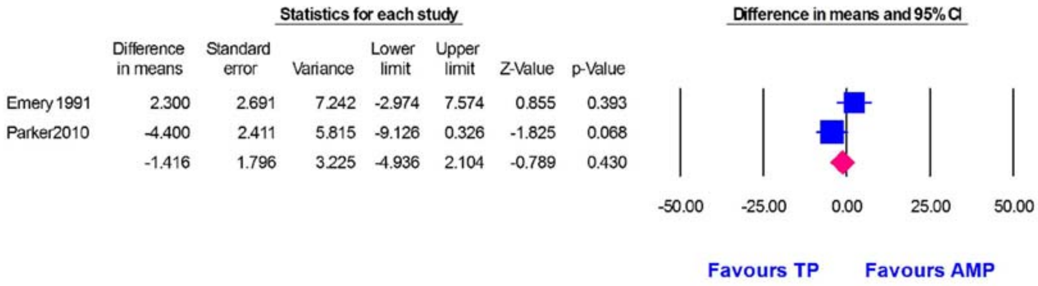

	Statistics for each study						
	Difference in means	Standard error	Variance	Lower limit	Upper limit	Z-Value	p-Value
Emery 1991	2.300	2.691	7.242	-2.974	7.574	0.855	0.393
Parker2010	-4.400	2.411	5.815	-9.126	0.326	-1.825	0.068
	-1.416	1.796	3.225	-4.936	2.104	-0.789	0.430

Figure A.7. Forest Plot of mean difference (MD) of hospital stay with 95% confidence interval, comparing between Thompson and Austin Moore groups.

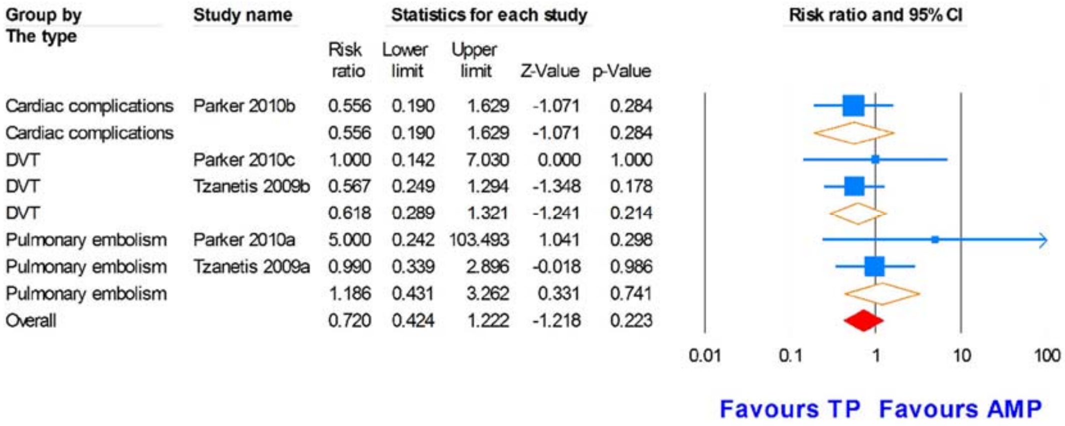

Group by The type	Study name	Statistics for each study				
		Risk ratio	Lower limit	Upper limit	Z-Value	p-Value
Cardiac complications	Parker 2010b	0.556	0.190	1.629	-1.071	0.284
Cardiac complications		0.556	0.190	1.629	-1.071	0.284
DVT	Parker 2010c	1.000	0.142	7.030	0.000	1.000
DVT	Tzanetis 2009b	0.567	0.249	1.294	-1.348	0.178
DVT		0.618	0.289	1.321	-1.241	0.214
Pulmonary embolism	Parker 2010a	5.000	0.242	103.493	1.041	0.298
Pulmonary embolism	Tzanetis 2009a	0.990	0.339	2.896	-0.018	0.986
Pulmonary embolism		1.186	0.431	3.262	0.331	0.741
Overall		0.720	0.424	1.222	-1.218	0.223

Figure A.8. Forest Plot of risk ratio (RR) of medical complications with 95% confidence interval, comparing between Thompson and Austin Moore groups.

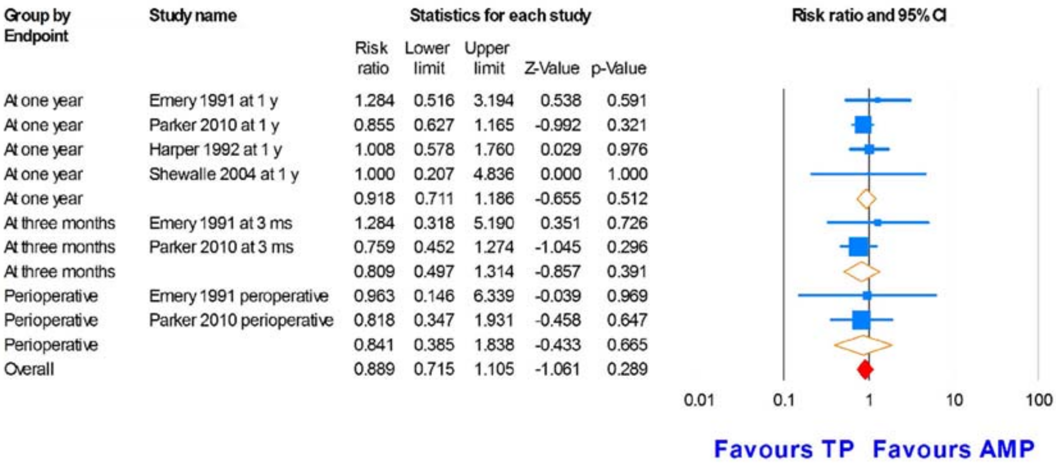

Group by Endpoint	Study name	Statistics for each study				
		Risk ratio	Lower limit	Upper limit	Z-Value	p-Value
At one year	Emery 1991 at 1 y	1.284	0.516	3.194	0.538	0.591
At one year	Parker 2010 at 1 y	0.855	0.627	1.165	-0.992	0.321
At one year	Harper 1992 at 1 y	1.008	0.578	1.760	0.029	0.976
At one year	Shewalle 2004 at 1 y	1.000	0.207	4.836	0.000	1.000
At one year		0.918	0.711	1.186	-0.655	0.512
At three months	Emery 1991 at 3 ms	1.284	0.318	5.190	0.351	0.726
At three months	Parker 2010 at 3 ms	0.759	0.452	1.274	-1.045	0.296
At three months		0.809	0.497	1.314	-0.857	0.391
Perioperative	Emery 1991 peroperative	0.963	0.146	6.339	-0.039	0.969
Perioperative	Parker 2010 perioperative	0.818	0.347	1.931	-0.458	0.647
Perioperative		0.841	0.385	1.838	-0.433	0.665
Overall		0.889	0.715	1.105	-1.061	0.289

Figure A.9. Forest Plot of risk ratio (RR) of mortality with 95% confidence interval, comparing between Thompson and Austin Moore groups.
